# A *Drosophila* Model Identifies a Critical Role for Zinc in Mineralization for Kidney Stone Disease

**DOI:** 10.1371/journal.pone.0124150

**Published:** 2015-05-13

**Authors:** Thomas Chi, Man Su Kim, Sven Lang, Neelanjan Bose, Arnold Kahn, Lawrence Flechner, Sarah D. Blaschko, Tiffany Zee, Gulinuer Muteliefu, Nichole Bond, Marysia Kolipinski, Sirine C. Fakra, Neil Mandel, Joe Miller, Arvind Ramanathan, David W. Killilea, Katja Brückner, Pankaj Kapahi, Marshall L. Stoller

**Affiliations:** 1 Department of Urology, University of California San Francisco, San Francisco, California, United States of America; 2 College of Pharmacy, Inje University, Gimhae, Republic of Korea; 3 The Buck Institute for Research on Aging, Novato, California, United States of America; 4 The Advanced Light Source, Lawrence Berkeley National Lab, Berkeley, California, United States of America; 5 Department of Medicine, Division of Nephrology, Medical College of Wisconsin, Zablocki VA Medical Center, Milwaukee, Wisconsin, United States of America; 6 Nutrition & Metabolism Center, Children’s Hospital Oakland Research Institute, Oakland, California, United States of America; 7 Eli and Edythe Broad Center of Regeneration Medicine and Stem Cell Research, University of California San Francisco, San Francisco, California, United States of America; 8 Department of Cell and Tissue Biology, University of California San Francisco, San Francisco, California, United States of America; 9 Cardiovascular Research Institute, University of California San Francisco, San Francisco, California, United States of America; National Cancer Institute, UNITED STATES

## Abstract

Ectopic calcification is a driving force for a variety of diseases, including kidney stones and atherosclerosis, but initiating factors remain largely unknown. Given its importance in seemingly divergent disease processes, identifying fundamental principal actors for ectopic calcification may have broad translational significance. Here we establish a *Drosophila melanogaster* model for ectopic calcification by inhibiting xanthine dehydrogenase whose deficiency leads to kidney stones in humans and dogs. Micro X-ray absorption near edge spectroscopy (μXANES) synchrotron analyses revealed high enrichment of zinc in the *Drosophila* equivalent of kidney stones, which was also observed in human kidney stones and Randall’s plaques (early calcifications seen in human kidneys thought to be the precursor for renal stones). To further test the role of zinc in driving mineralization, we inhibited zinc transporter genes in the ZnT family and observed suppression of *Drosophila* stone formation. Taken together, genetic, dietary, and pharmacologic interventions to lower zinc confirm a critical role for zinc in driving the process of heterogeneous nucleation that eventually leads to stone formation. Our findings open a novel perspective on the etiology of urinary stones and related diseases, which may lead to the identification of new preventive and therapeutic approaches.

## Introduction

Calcification processes are central for many aspects of development including bone and tooth growth [1,2]. In contrast, ectopic calcification is the accumulation of mineralized tissue occurring in a dysregulated fashion [3] that leads to disease states, including urinary stone disease [4]. However, initiators of calcification are poorly understood, limiting preventive and therapeutic approaches for nephrolithiasis. Although calcium hydroxyapatite is thought to serve as a nidus for many mineralized deposits and structures across phyla and species [5–8], the precursor pathways leading to the presence of hydroxyapatite are poorly characterized. Thus far, only a small number of genes have been linked to kidney stones, reflective of the limitations of current mammalian model approaches to understand their effects on the calcification process. While murine and porcine models have been utilized [9,10], the relatively longer time for onset of stone formation and lack of genetic tools have limited genetic screening as a means of exploring mineralization.

To overcome this issue, insect physiologists have proposed the use of invertebrate models for advancing the understanding of mechanisms by which mineralization occurs [11,12]. Two models of kidney stone disease utilizing *Drosophila melanogaster* [13,14] have been published, providing a precedent for the use of invertebrate models in understanding mineralization. These models, however, relied on exogenous exposure of flies to substances including ethylene glycol and oxalate to initiate the formation of calcified particles. Their consumption in high levels is not a normal part of the fly diet. In contrast, we utilized a genetic approach, silencing xanthine dehydrogenase (*Xdh*) to apply *Drosophila melanogaster* as a model to study the mechanisms by which ectopic calcification occurs. This model allows exploration of the complex interplay between proteins, minerals, genes, and environmental exposures which are known to influence kidney stone formation [15].

*Drosophila* Malpighian tubules are the functional equivalent of the human kidney convoluted tubules. Congruent to the human renal tubule, they are the site of solute transport and excretion of calcium, uric acid, and phosphorus [16]. Intraluminal mineralized particles within the Malpighian tubule termed concretions by insect physiologists have been described, thought to serve as sites for solute deposition [17]. We analyzed *Drosophila* Malpighian tubule specimens in parallel with human renal tissue utilizing advanced synchrotron radiation-based techniques to confirm that fly concretions shared characteristics commonly seen in ectopic kidney calcification plaques as well as kidney stones. Intriguingly, we found that all tissue samples contained significant, non-trace amounts of zinc (Zn). To better understand the significance of Zn in these tissues, we genetically inhibited Zn transport and found that this led to markedly decreased accumulation of calcified concretions within the fly tubule. These findings were corroborated by functional analyses in which Zn levels were altered using dietary and pharmacological manipulations, demonstrating a functional connection between the levels of Zn and increased concretion formation. Our data supports the idea that Zn facilitates calcification and represents a possible target for developing preventive and therapeutic strategies against nephrolithiasis.

## Results

### Inhibition of xanthine dehydrogenase results in fly stones within the *Drosophila* Malpighian tubule

**Fig 1:**
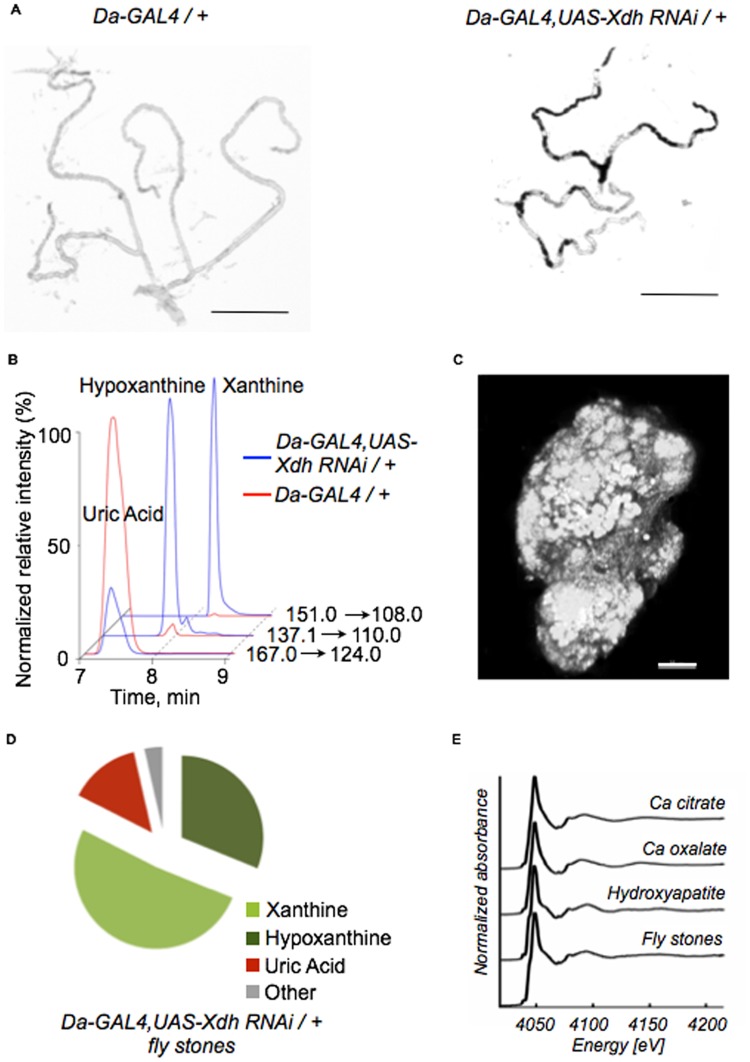
Inhibition of xanthine dehydrogenase leads to ectopic calcification in the fly Malpighian tubules. (A) Representative tubule images taken from control flies (*Da-GAL4/+*, left panel) and flies upon *Xdh* knockdown (*Da-GAL4*, *UAS-Xdh RNAi /+*, right panel). Concretions are dark and intraluminal. Scale bars: 500 μm. (B) HPLC-MS (multiple reaction monitoring) analysis of whole flies demonstrates significant reduction in uric acid levels in *Da-GAL4*, *UAS-Xdh RNAi /+* flies compared to control flies with concomitant increase in xanthine and hypoxanthine levels. (C) Confocal microscopy of a fly tubule concretion isolated after *Xdh* knockdown. The gross appearance resembles that of a miniaturized kidney stone. Scale bar: 20 μm. (D) HPLC-MS (multiple reaction monitoring) analysis of concretions taken from these *Da-GAL4*, *UAS-Xdh RNAi /+* flies demonstrates that they primarily contain xanthine and hypoxanthine with smaller amounts of uric acid and other purine metabolites also present. (E) μXANES demonstrates the presence of hydroxyapatite as the primary calcium salt in *Drosophila* concretion samples.

Seeking a *Drosophila* model for urinary stone disease, we examined the consequences of knocking down orthologs of human genes implicated in kidney stone formation on mineralized concretion formation in adult *Drosophila* Malpighian tubules. From a screen of ten such genes we observed a strong incidence of concretion formation upon inhibition of xanthine dehydrogenase (*Xdh*). Human mutations in xanthine dehydrogenase activity result in the disorder xanthinuria type I. An equivalent disease also occurs in dogs, most notably the Cavalier King Charles spaniel breed [18]. Patients with xanthinuria type I, one of the few single gene disorders known to result in nephrolithiasis in humans, form debilitating, recurrent kidney stones [19]. We observed that RNAi inhibition of *Xdh* [20] resulted in significantly increased tubule concretion formation when compared to controls (Fig 1A). RNAi knockdown efficiency was confirmed with RT-PCR (S1 Fig). Under light microscopy examination, concretions were visible as dark intraluminal contents within the Malpighian tubule and had the appearance of small stones. Upon dissection they also looked like small stones and their hardness could be felt between ones’ fingers. Given their nature, we refer to these exuberant concretions as fly stones. To confirm that this fly stone accumulation phenotype was specific to the gene function and not due to strain background, stone presence was confirmed in a second RNAi line against *Xdh* (S2 Fig). Next, using high performance liquid chromatography (HPLC)—mass spectrometry (MS) (multiple reaction monitoring, MRM)-based targeted metabolomics for quantification of purines, we found that RNAi knockdown of *Xdh* indeed increased xanthine and hypoxanthine, and decreased uric acid (Fig 1B) levels in the whole fly, supporting the proposed biochemical role of *Xdh*. Furthermore, we also observed that allopurinol, a pharmacologic inhibitor of xanthine dehydrogenase activity, led to stone formation (S3 Fig). Thus, both genetic and pharmacological inhibition of xanthine dehydrogenase led to stone formation in *D*. *melanogaster*.

Fly stones in our model were physically and visually similar to human kidney stones, though smaller in size (Fig 1C). To analyze the chemical nature of these fly stones, we first performed Fourier Transform Infrared Spectroscopy (FTIR), the traditional method by which kidney stones are analyzed clinically, and confirmed that these fly stones contained xanthine (S4 Fig). Targeted metabolomics demonstrated, however, that these concretions not only contained xanthine, but also hypoxanthine (Fig 1D), the upstream metabolite we found elevated in whole fly specimens upon knockdown of *Xdh*. To further ascertain that the fly stones represented mineralized tissue, we examined if they contained cellular debris by assessing the presence of nucleotides. DAPI-stained control samples verified that fly stones samples were relatively free of cellular debris as a possible contaminant source (S5 Fig). Historically, patients with xanthinuria type I are thought to have stones made of pure xanthine resulting from spillage of excess xanthine into the urinary space. However, based on the stone-like appearance of these fly concretions, we hypothesized that these concretions contain components commonly associated with ectopic calcification. Micro X-ray absorption near edge spectroscopy (μXANES) permits interrogation of the chemical environment within a sample point of interest with sub-micron resolution. Utilizing μXANES analysis, we demonstrated the presence of hydroxyapatite in these fly stones (Fig 1E). The presence of hydroxyapatite within these concretions suggested that *Drosophila* fly stone formation may bear similarities to human ectopic calcification since hydroxyapatite is a fundamental building block for human kidney stones [21]. Our results show that *Xdh* suppression leads to the accumulation of xanthine and hypoxanthine. The fly stone phenotype with the presence of hydroxyapatite provided an invertebrate model to study the process of *in vivo* mineralization.

### Comparative analysis of fly and human calcified plaques suggests a role for zinc in ectopic calcification

**Fig 2:**
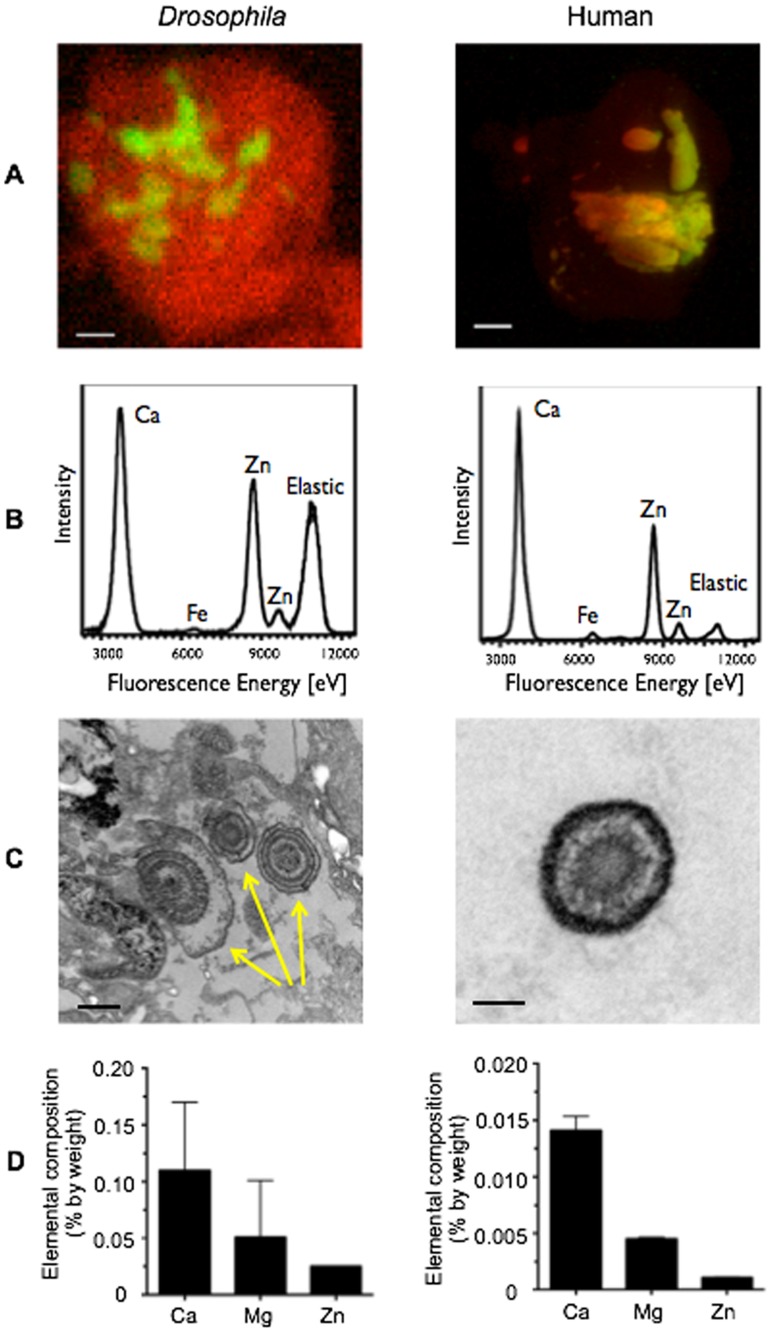
*Drosophila* concretions, human Randall’s plaques, and human kidney stones show enrichment of zinc. (A) Micro-X-ray fluorescence (μXRF) maps of *Da-GAL4*, *UAS-Xdh RNAi /+* concretions (left panel, scale bar: 10 μm) and human Randall’s plaques (right panel, scale bar: 100 μm) demonstrate the presence of zinc in red and calcium in green. (B) μXRF elemental analysis of the samples from (A) demonstrate similar elemental composition for both *Da-GAL4*, *UAS-Xdh RNAi /+* concretions (left panel) and human Randall’s plaques (right panel), including the presence of calcium (Ca), iron (Fe), and zinc (Zn). (C) Transmission electron microscopy imaging of concretions in the lumen of the Malpighian tubule demonstrates the presence of ring-like structures, as indicated by the yellow arrows (left panel, scale bar: 500 μm). Ring structures with homologous appearance are seen in Randall’s plaques taken from a human renal papilla biopsy material (right panel, scale bar: 100 μm) when imaged in a similar fashion. (D) ICP-OES analysis of pooled fly concretion samples from 300 dissected tubule specimens demonstrates the presence of calcium (Ca), magnesium (Mg), and zinc (Zn) (left panel, n = 2 biological replicates). These levels are mirrored in human xanthine stone samples (right panel, n = 2 biological replicates). Data shown are the mean ± SEM.

*Drosophila* fly stones were analyzed using micro X-ray fluorescence (μXRF) mapping to determine their elemental compositions. μXRF mapping at 11keV with 15 micron / pixel and 2.5 micron / pixel resolution demonstrated the consistent presence of both calcium (Ca) and Zn in each specimen (Fig 2A). μXRF spectra recorded at 14 keV of randomly selected regions of the samples demonstrated an abundance of Ca and Zn, with trace amounts of iron (Fig 2B).

To compare *Drosophila* fly stones to human calcified tissue, human renal papilla biopsies containing visually apparent Randall’s plaque material were used for comparative analysis. These plaques, residing in the tip of the renal papilla, are calcified structures thought to be precursor lesions for calcium-based kidney stones [22,23]. μXRF mapping and spectral analysis demonstrated that, like fly stones, Randall’s plaques contain consistent, abundant amounts of both Ca and Zn (Fig 2A and 2B). Transmission electron microscopy imaging revealed the presence of small spherical structures composed of electron dense and relatively lucent layers in both fly stones and Randall’s plaques (Fig 2C). These layered structures have been similarly demonstrated previously [11,24]. Interestingly, these spherules also have been observed in electron microscopic images of developing bone, atherosclerotic plaques, and other forms of ectopic calcification [25,26] and may represent a universal building block in calcification processes shared across species [27,28]. Taken together, these data demonstrated significant structural and elemental similarity between *Drosophila* fly stones and human Randall’s plaques.

To quantitatively compare the mineral components of fly concretions from specimens after *Xdh* inhibition and human xanthine stones, we used inductively coupled plasma optical emission spectroscopy (ICP-OES). This analysis revealed Ca, magnesium (Mg), and Zn as the major metal elements present in each specimen (Fig 2D). In fly stones, each metal composed 0.11±0.06, 0.051±0.05, and 0.025±0.0001% by weight respectively and in the human stone samples, 0.014±0.001, 0.0045±0.0001, and 0.0011±0.0001%. The amount of each metal present as a relative ratio to one another appears to be preserved between the two samples, though their absolute amounts differ. The difference in absolute amount of metal between the human and fly samples might be expected given the higher complexity of human plaque content, which includes a significant amount of protein and small molecules. Interestingly, Zn was consistently present in three different mineralized tissues across two distinct species (fly stones, human Randall’s plaques, and human xanthine kidney stones). Next we examined the hypothesis that modulating Zn levels should alter ectopic calcification and tested this hypothesis in the fly tubule.

### Genetic modulation of zinc levels alters *Drosophila* stone formation

We examined the role of Zn in initiating fly stone formation in *Drosophila* using a genetic approach to alter zinc transport and availability. ZnT family transporters are a group of transmembrane proteins, over 20 of which have been characterized in humans [29]. In *Drosophila*, three ZnT transporters (*CG3994*, *CG11163*, and *CG17723*) have been reported to be highly expressed in the Malpighian tubule whose function is to regulate Zn movement across the cell membrane [30,31].

**Fig 3:**
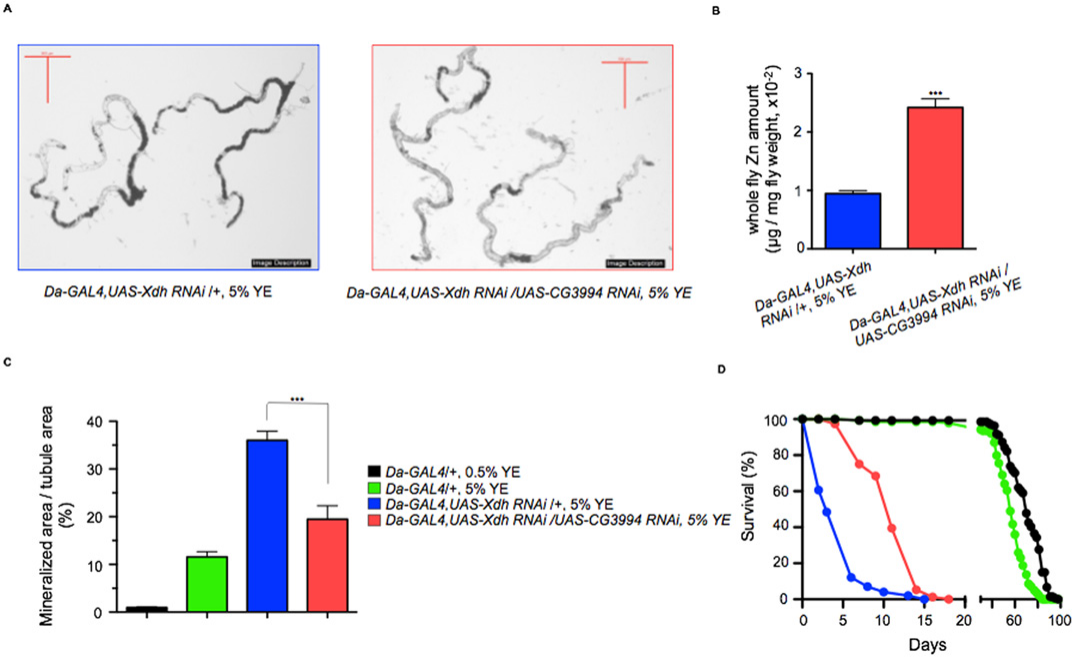
Inhibition of zinc transporters inhibits concretion formation in a fly model for concretion formation. (A) Gross photomicrographs of Malpighian tubule concretions isolated from *Da-GAL4*, *UAS-Xdh RNAi /+* flies fed on a 5% YE diet (left panel) demonstrate significant reduction in concretion formation after simultaneous inhibition of the zinc transporter *CG3994* (right panel). Scale bars: 500 μm. (B) Upon simultaneous RNAi inhibition of xanthine dehydrogenase and CG3994, male flies were fed 5% YE. After 2 days, flies were anesthetized with CO_2_, their body weights measured and whole flies homogenized and analyzed with ICP-OES. Whole-fly levels of Zn were significantly higher after zinc transport inhibition (red bar) compared to flies expressing *Xdh* inhibition alone (blue bar, ***p<0.001, Student’s t-test, n = 3). Data shown are the mean ± SEM. (C) Measuring the area of the tubule lumen occupied by mineralized concretions, *Da-GAL4 /+* control flies produce significantly more concretions after feeding 5% YE compared to 0.5% YE (green versus black bars). With silencing of *Xdh*, tubule concretion production increases dramatically (blue bar), but simultaneous inhibition of xanthine dehydrogenase and *CG3994* resulted in significant reduction in concretion accumulation compared to *Da-GAL4*, *UAS-Xdh RNAi /+* flies (red bar) (***p <0.001, one way ANOVA with Bonferroni post-hoc test, n = 14–43). (D) Similarly, while *Da-GAL4*, *UAS-Xdh RNAi /+* flies exhibit significantly reduced survivorship (blue line), simultaneous inhibition of *CG3994* with xanthine dehydrogenase rescued survivorship when compared with *Da-GAL4*, *UAS-Xdh RNAi /+* animals on 5% YE (red line, logrank test, n = 76–97).

In our model, the presence of large, obstructing Malpighian tubule stones shortened the survival of adult flies. Inhibition of *Xdh* conferred a median lifespan of only 3 days when flies were fed a high yeast diet (Fig 3D) compared to the control lifespan of around 60 days. We first examined whether survivorship was affected by silencing Zn transporters. After confirming Zn transporter RNAi knock-down efficiency (S1 Fig), we simultaneously inhibited each *Drosophila* ZnT transporter and *Xdh*. This was performed by crossing each ZnT transporter RNAi line with a strain harboring the *Xdh* RNAi transgene and the ubiquitously expressing *Daughterless(Da)-GAL4* driver. Knock-down of all three ZnT transporters significantly rescued survivorship (Fig 3D and S6 Fig), though the effect was strongest with *CG3994*, which extended median lifespan from 3 to 12 days (p<0.001). To verify the effect of reducing Zn transport on fly stone formation, the degree of mineralization was quantified by calculating the percentage of the lumen occupied by stones. We found that concurrent inhibition of *Xdh* with all ZnT transporters significantly reduced fly stone formation when compared with *Xdh* silencing alone (Fig 3A–3C and S6 Fig). *CG3994* conferred a reduction of the tubular luminal space occupied by fly stones from 36.1±1.9 to 19.5±2.8% (p<0.001), highlighting the critical role of Zn in the mineralization process.

Of the three ZnT transporters, *CG3994* inhibition provided the most significant fly stone reduction and survivorship rescue (Fig 3C and 3D and S6 Fig). *CG3994* functions as a Zn-specific transporter primarily expressed in the Malpighian tubule where it is located on the apical membrane of the tubular cells and facilitates the excretion of Zn across the tubular cell membrane and into the lumen. Zn efflux from the tubule lumen is mediated by *CG3994* and its overexpression has been shown to induce resistance to zinc toxicity, which occurs at high levels of dietary ingestion in *Drosophila* [31]. Based on these previously published data, we postulated that by inhibiting this gene’s function, Zn should be sequestered away from the tubule lumen where it would otherwise be excreted, and should therefore accumulate at higher levels in the fly. Consistent with this idea, ICP-OES analysis revealed that simultaneous *Xdh* and *CG3994* knockdown led to an increase in whole-fly Zn levels (Fig 3B). This effect was selective for Zn transport since other metal levels remained unchanged (S7 Fig). These data demonstrating an increase in whole-fly Zn levels were consistent with our hypothesis that *CG3994* was affecting concretion formation by preventing Zn movement into the tubular lumen. Our findings corroborated the idea that ZnT transporters play an important role in facilitating Zn tubule excretion, and that Zn excretory reduction diminishes intraluminal mineralization.

To concurrently test whether other factors might affect the mineralization process more strongly than Zn upon inhibition of *Xdh* we conducted an RNAi-based suppressor screen to identify alternative modifiers of ectopic calcification. A biased screen of 25 candidate genes whose function was suspected to relate to mineral transport in the Malpighian tubule was performed [32]. Since fly stone formation was associated with reduced survivorship (Fig 3C and 3D and S6 Fig), we utilized survivorship as a high-throughput assay tool to rapidly identify candidate genes that suppressed stone formation. Our recombinant *Da-GAL4*, *UAS-Xdh RNAi /+* fly (median survival 3 days and tubular lumen stone formation of 36.0±1.9% on high yeast food) was crossed with candidate RNAi strains for this screen. RNAi lines conferring a three-fold rescue in survivorship to a median lifespan of 9 days were then dissected to confirm whether inhibition of the fly stone-forming phenotype was also present. This screen identified multiple suppressors, including *mtrm* (median survival 9 days, tubular lumen stone formation 26.1±3.95%) and *Cp1* (median survival 10 days, tubular lumen stone formation 30.4±2.7%). These genes have been characterized as meiosis and autophagy regulators respectively [33,34]. *CG3994*, however, remained the most potent suppressor of fly stone formation and also conferred the greatest survivorship rescue. These results further emphasize the importance of Zn over other metals in the initiation of fly stone formation process.

### Dietary and pharmacologic modulation of zinc levels alter *Drosophila* stone formation

**Fig 4:**
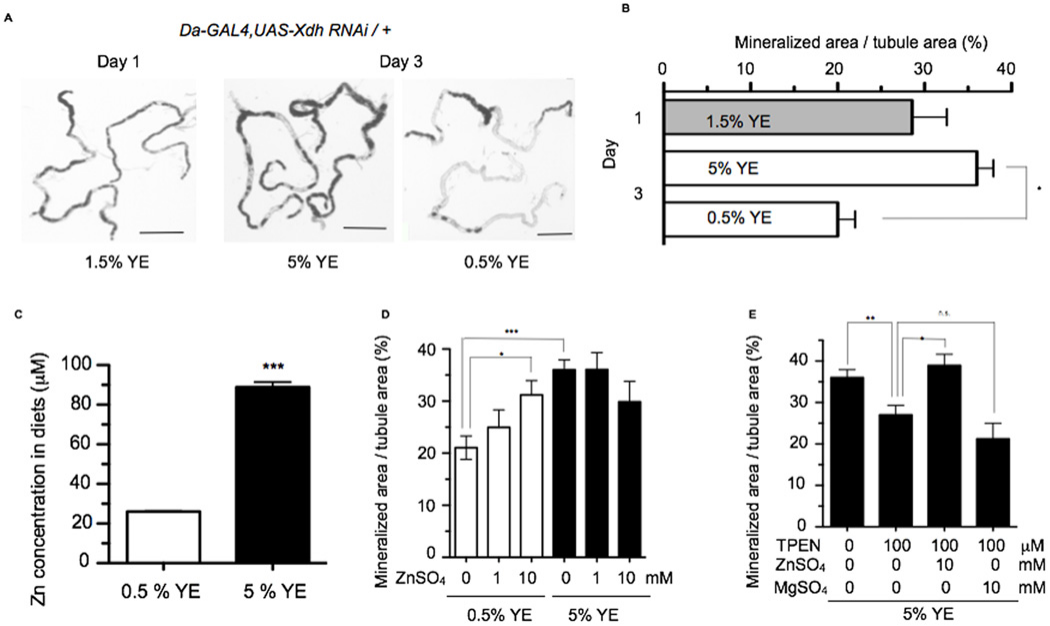
Zinc modulates stone formation in a fly model for ectopic calcification. (A) Typical tubule images taken from *Da-GAL4*, *UAS-Xdh-RNAi /+* flies at eclosion (Day 1, 1.5% YE) and after feeding 2 days on high yeast (Day 3, 5% YE) and low yeast (Day 3, 0.5% YE) diets. (B) The extent of tubular mineralization is quantified as the percentage of the tubule lumen occupied by mineralized material (*p <0.001, Student’s t-test, n = 14–48). Scale bars: 500 μm. Data shown are the mean ± SEM. (C) The amount of Zn in the components of a standard *Drosophila* diet (corn meal, yeast, sugar and agar) was measured with ICP-OES for both 0.5% YE and 5% YE diets. 0.5% YE is comprised of corn meal 8.5 g, yeast 0.5 g, sugar 5 g, and agar 0.46 g in 100 ml deionized distilled water. 5% YE is composed of the same components as 0.5% YE with the exception of 5 g yeast added instead of 0.5 g yeast (***p<0.001, student t-test, n = 3). (D) *Da-GAL4*, *UAS-Xdh RNAi / +* flies fed on 0.5% YE and 5% YE diets were supplemented with different doses of Zn. This led to a dose-dependent increase in accumulation of concretions on 0.5% YE feeding (*p <0.05, ***p <0.001, one way ANOVA with Bonferroni post-hoc test, n = 10–48). Zn supplementation had no effect on altering concretion formation in 5% YE-fed animals, which may reflect a saturation of the calcification process under these conditions. (E) Supplementation of 5% YE diet with the zinc chelator TPEN reduced concretion formation. This effect could be reversed with the addition of 10 mM Zn but not 10 mM Mg (*p <0.05, **p <0.01, one way ANOVA with Bonferroni post-hoc test, n = 10–43). Data shown are the mean ± SEM.

Dietary and pharmacologic approaches that alter Zn availability were used to explore the physiologic significance of Zn in concretion formation. As ectopic calcification in urinary stone formation and other calcification-based diseases is in part affected by diet [35–37], we examined whether stone formation in *Drosophila* could be further exacerbated with dietary change. *Xdh*-silenced flies formed significantly less concretions after being transferred to a low yeast (yeast being the primary source of dietary protein for the fly) diet (0.5% yeast extract (YE)) compared to a high yeast diet (5% YE) (Fig 4A and 4B). Following *Xdh* knockdown, low yeast feeding resulted in a 45% decrease (p<0.001) in Malpighian tubule concretion formation compared to high yeast feeding (Fig 4B). ICP-OES analysis demonstrated that the dietary Zn content of high yeast food was four times that of low yeast food (Fig 4C). To test whether Zn alone could replicate the effects of high yeast feeding, it was added to 0.5% YE food. Supplementing the low yeast diet with 1 mM and 10 mM ZnSO_4_ yielded increased fly stone formation in a dose-dependent fashion compared to a low yeast diet without Zn supplementation. Zn supplementation, however, did not increase fly stone formation beyond the effects of high yeast feeding alone (Fig 4D), likely due to the ceiling effect.

Bioavailable Zn was reduced using a pharmacological approach to confirm the central role of Zn in the mineralization process. Dietary supplementation with N,N,N’N’-tetrakis-(2-pyridylmethyl)ethylenediamine (TPEN), a zinc-selective chelator [29,38], reduced fly stone formation in flies fed a high yeast diet after *Xdh* inhibition (Fig 4E). The maximal non-lethal suppressive dose of TPEN for inhibiting fly stone formation was 100 μM (S8 Fig). The protective effect of TPEN could be overcome with supplemental Zn. Mg, another cation, had no effect (Fig 4E), demonstrating the specificity of TPEN for Zn on the fly ectopic calcification process in the Malpighian tubule. Taken together, these data demonstrate a role for Zn in modulating mineralization.

## Discussion

### A genetic *Drosophila* model to study ectopic calcification

Up to 12% of Americans will experience a symptomatic kidney stone [39] each year and as many as 50% of these patients will require surgical intervention [40]. Nephrolithiasis is a significant source of morbidity and mortality [41–44]. In contrast to the major advancements in the surgical management of this disease over the last few decades, our understanding of the underlying mechanisms of stone formation and development of effective prophylactic medical management has seen little progress over the same time period [45,46]. A major barrier to such progress includes a rudimentary understanding of the genetic and environmental underpinnings of ectopic calcification, the pathologic mineralization process leading to urinary stone formation. To help identify such factors we selected the *Drosophila melanogaster* Malpighian tubule system as a model for nephrolithiasis. Previous work has demonstrated that *Drosophila* can be used as a model for kidney stones; these groups relied on dietary supplementation of non-physiologic, toxic substances to achieve stone formation—namely ethylene glycol and its metabolite, oxalate [13,14,47]. Our work complements previous work by identifying a genetic means to modulate fly mineralization.

It is important to consider the physiologic nature of concretion accumulation. Dube et al [48] demonstrated that flies mainly form concretions in the anterior Malpighian tubules and postulated that this was a means to regulate calcium; tubular calcium excretion was thought to be a normal homeostatic function in the wild type fly. These concretions were visible as small calcium-containing spherules. In our model, similar appearing concretions are present. However, in the pathologic phenotype that appears to be a direct result of *Xdh* gene silencing, the rapid accumulation of tubular concretions, both in large quantities and increased size, results in tubular obstruction and dilation of the Malpighian tubule and appears to be responsible for premature death (Fig 1A). Concretions may lead to the formation of these larger mineralized particles that we term fly stones. We believe these represent two distinct phenomena, the latter being one of pathologic, ectopic, and exuberant calcification while the former is a normal, physiologic process. The pathologic fly stone is the foundation upon which our model is built.

Inhibition of *Xdh* led to a shortened lifespan which was used to rapidly identify modulators of fly stone formation with the presumption that this excessive ectopic calcification process was pathologic in nature and harmful to the fly. The reduced lifespan appears to be due to the presence of excessive, enlarged, ectopic, and consistently obstructive fly stones (Fig 3C and 3D). Many genes likely work via multiple pathways that modulate both concretion formation and survivorship independent of one another. Thus, shortened lifespan may not be a direct result of obstructive fly stone formation. Inhibition of *CG3994* alone led to only a modest extension of lifespan (S9 Fig) and is therefore unlikely to account for the dramatic four-fold increased survivorship seen with zinc transport inhibition in the background of *Xdh* suppression. Modulating genes known to extend lifespan (including inhibition of target of rapamycin (TOR) and insulin signaling pathway genes [49]) did not suppress concretion formation upon *Xdh* silencing (data not shown). Thus, the rescue of fly stone formation appears to be a specific phenotype that is not significantly altered by pathways determining longevity. Normal physiologic concretion formation varies over the course of a fly’s lifespan (S2 Fig) and likely even over the course of the day [48]. To attempt to control for this physiologic aspect of concretion formation dissections were performed at the same time of day for each experiment and at the same day of life for each condition. Day of life 3 was selected to compare concretion accumulation between different conditions as flies died rapidly upon *Xdh* inhibition soon after that point under high yeast feeding conditions.

### The role of xanthine dehydrogenase in calcification

Xanthine dehydrogenase catalyzes the final steps of purine degradation [50]. In humans, xanthine and xanthine dehydrogenase are linked to both atherosclerosis and urinary stones, two forms of ectopic calcification [51,52]. Ectopic calcification was studied in the *Drosophila* Malpighian tubule utilizing a background of inhibition of *Xdh* [53]. Xanthine dehydrogenase is deficient in a subset of patients with nephrolithiasis [19] and some dog species [18]. The demonstration of ectopic calcifications in *Drosophila* deficient in *Xdh* reflects a conserved role for xanthine dehydrogenase in ectopic calcification. Parallels of this model with human urinary stone disease are further illustrated by the exacerbation of *Drosophila* stone formation by environmental factors such as a high yeast (high protein) diet and similarities in the elemental composition between fly stones, human Randall’s plaques, and human xanthine kidney stones. *Xdh* deficiency in humans is known to lead to accumulation of xanthine in the urine which has been traditionally considered the cause of urinary stone formation in these patients [19]. Our results in *Drosophila* recapitulate these findings, and demonstrate a conserved role for xanthine and hypoxanthine in enhancing calcification in the fly tubular lumen.

### Zinc plays an important role in calcification

While one might have predicted the accumulation of xanthine stones in an *Xdh* inhibition model, the concurrent presence of zinc was quite unexpected. Zinc’s presence in human xanthine stones confirmed that these stones are not a pure accumulation of xanthine as presumed since this disease was first described [19]. Remarkably, zinc was also present in human Randall’s plaques from a patient who had calcium oxalate, and not xanthine stones. In human xanthinuria type I, Randall’s plaques are not typically present as precursor lesions for stones. Calcium oxalate stones are the most common type of nephrolithiasis treated worldwide. The presence of non-trace levels of zinc in fly stones, human xanthine stones and calcium oxalate plaques highlights the potential role of zinc in the ectopic calcification process. Its consistent presence supports the role of heterogeneous nucleation in stone formation.

In our fly model, the ectopic calcification phenotype was rescued by reducing zinc in the Malpighian tubule. We demonstrated that altering zinc levels affected fly stone formation by changing dietary zinc ingestion, applying a pharmacologic zinc chelator, and inhibiting three different ZnT transporters. All three of these transporters (*CG3994*, *CG11163*, and *CG17723*) significantly reduced the formation of fly stones (S6 Fig). All three of them are expressed in the Malpighian tubule, though *CG11163* and *CG17723* are also expressed elsewhere at significant levels, including the gut and salivary gland respectively [54,55]. Of the three ZnT transporters, only *CG3994* is almost exclusively expressed in the Malpighian tubule [31]. For *CG11163* and *CG17723*, their activity in other tissues might account for a change in both whole body Zn levels and changes in stone formation. However, *CG3994* inhibition alone led to the strongest stone rescue phenotype. This transporter, primarily expressed in the Malpighian tubule, confirms that this phenotype change is likely due to Zn transport occurring at the level of the tubule itself since *CG3994* inhibition alone was enough to reduce the stone formation phenotype. Inhibiting all three transporters simultaneously would be one approach for confirming the importance of zinc in the stone formation process, but zinc is also important for a number of physiologic functions. Interfering with normal Zn trafficking to too great of an extent has lethal consequences in *Drosophila* [54] and therefore taking a combined approach to inhibiting several ZnT transporters at once to test their relative importance in the stone formation process was not possible.

The central role for Zn in ectopic calcification may have far-reaching implications for other diseases with ectopic calcifications including atherosclerosis [56], and neurodegenerative disorders [57]. For example, in microcalcifications of early atherosclerotic plaques, zinc has been co-localized to areas of high calcium concentration that contain hydroxyapatite [58]. Our findings suggest that Zn likely plays a critical role in initiating the mineralization pathway leading to calcification since hydroxyapatite is thought of as an early nidus for biomineralization [59]. As such, zinc may be targeted at a fundamental level to treat a number of ectopic calcification-related diseases.

Large scale epidemiological studies have associated zinc with increased risk for nephrolithiasis [60], but these studies have not defined a causal role for zinc in mineralization. Our synchrotron-based analyses demonstrated that Zn is closely associated with hydroxyapatite, a known early nidus for the mineralization process. This suggests that Zn may facilitate or modulate the binding between calcium hydroxyapatite and other components of calcified plaques or concretions. Alternatively, it may provide a scaffold to which hydroxyapatite attaches itself to ultimately form a mineralized plaque.

Randall’s plaques are thought to represent the early stages of human renal stone formation, and our findings demonstrated that distinct areas within these plaques are highly enriched in zinc. Similarly, we propose that *Drosophila* stones, which form over the course of days, represent early, rather than late, events in the cascade culminating in calcification. Zinc’s presence at this early stage of ectopic calcification in two different species as well as its close association with hydroxyapatite suggest a critical primary role in the initiation of calcification, rather than a secondary role as has been proposed previously [61]. This is further supported by the functional effects of zinc on *Drosophila* stone formation. Genetic and pharmacological approaches reduced the intraluminal availability of Zn. *CG3994* is known to facilitate transport of Zn into the tubular lumen. Inhibition of *CG3994* as well as Zn chelation with TPEN in our model dramatically reduced fly stone formation. Zn thus appears to have a central, functional role in the initiation of stone formation.

### Modulating zinc homeostasis as a therapeutic strategy to prevent ectopic calcification

Many forces are at work balancing normal from ectopic calcification processes [62], and a better understanding of this interplay can lead to new therapeutic strategies. Thus, manipulation of Zn could be leveraged as a therapeutic target for the prevention of many forms of ectopic calcification. BLASTP searches show that the protein encoded by *CG3994* shares homology with human ZnT1 (25.6% identity and 43.1% similarity). This level of homology between *CG3994* and human ZnT1 provides some evidence that a similar type of zinc transport may be occurring at the level of the tubule in both *Drosophila* and humans since *CG3994* and ZnT1 are known to be expressed in the fly Malpighian tubule and human kidney respectively. Our findings in *Drosophila* may therefore bear translation to human physiology. However, since serum zinc levels in humans are tightly regulated [63] and are critical for many physiologic processes including regulation of oxidative stress [64] and development [65] it may be the local regulation of zinc rather than whole body zinc levels that are important. To date no evidence supports the use of zinc supplementation or restriction as a treatment for the prevention of urinary stones, likely due to the tight local regulation of zinc levels that prevents dietary changes from affecting serum and organ levels to a significant extent. Optimal regulation of local zinc concentrations in specific cellular compartments and tissues may hold the key to leveraging zinc as a therapeutic target for disease treatment and prevention. With ectopic calcification linking so many diseases together, translating the clinical benefits of targeted zinc therapy may be applicable to many human illnesses. Drugs such as metal chelators may prove to be a novel means of suppressing the process of ectopic calcification. Future studies will evaluate whether this strategy can be applied effectively for therapeutic development.

## Materials and Methods

### Human sample collection

In accordance with the ethical guidelines set forth by PLoS ONE, for human Randall's plaque samples utilized in this study, an appropriate institutional human research protocol was submitted at the University of California, San Francisco, and approval was obtained (study #11–06444). Since these samples were taken as normal course of surgery, we obtained an institutionally approved consent waiver for the purposes of this study. For xanthine stones, the committee on human research at the Children's Hospital of Philadelphia (from which they were obtained) deemed that no Institutional Review Board (IRB) approval or patient consent for sample collection was necessary since these stones would normally be discarded.

### Candidate gene *in vivo* RNAi screen for concretions in the Malpighian tubules

We established a *Drosophila* nephrolithiasis model by using the RNAi line for *Xdh* (*CG7642*, VDRC transformant ID 25175), driven by *Da-GAL4*. For RNAi screening, we generated the recombinant line *Da-GAL4*, *UAS-Xdh RNAi /Tm6*. Each transgenic fly strain containing inducible UAS-hairpin RNA elements on their chromosomes towards a single coding protein sequence was obtained from the Vienna *Drosophila* RNAi Center, Vienna (VDRC), Austria. Virgin *Da-GAL4*, *UAS-Xdh RNAi /Tm6* flies were crossed into each transgenic male fly, then progeny were sorted on the day of eclosion and transferred to low and high yeast diets. Tubules from knockdown candidate flies were dissected and concretion formation determined on day 3 after sorting.

### Fly husbandry and dietary manipulations

Flies were maintained on standard fly yeast extract medium (1.5% YE) containing 1.5 g of yeast, 5 g of sucrose, 0.46 g of agar and 8.5 g of corn meal and 1.5% tegosept in 100 ml distilled water. For high yeast (5% YE) and low yeast (0.5% YE) diets, the amount of yeast was adjusted to 5 g and 0.5 g, respectively.

### Survival analyses

For survivorship analysis, fly survival was scored while flies were transferred to fresh media every 2–3 days and the significance of change was measured using the log-rank test as previously described[49]. All flies were obtained or purchased from the Bloomington *Drosophila* Stock Center (Indiana, IN) and VDRC (Vienna, Austria) unless otherwise mentioned. Flies were kept in a temperature (25°C) and humidity (60%) controlled designated fly room with a 12-hour light / dark cycle.

### Malpighian tubule dissection

For dissection and imaging assays, upon *Xdh* knockdown, flies were transferred from stock food to diets dictated by the experiments (e.g, high or low yeast, presence or absence of Zn chelators). After two days, adult male flies were dissected and their tubules imaged for the presence of concretions. Flies were anesthetized by CO_2_ on standard flypads (Cat # 59–108, Genesee Scientific, San Diego, CA) and fly tubules were dissected under a dissecting light microscope (SZ61, Olympus, Center Valley, PA) on a culture dish in droplets of Schneider’s *Drosophila* medium (Cat # 11720–034, Invitrogen, Carlsbad, CA), utilizing fine forceps (Roboz ceramic, Gaithersburg, MD). Tubules were imaged utilizing a Leica M165 FC microscope with Leica Application Suite V3 analyzing software (Buffalo Grove, IL).

### Quantification of tubule mineralization

The level of concretion accumulation was quantified using the public domain ImageJ software (http://rsb.info.nih.gov/ij). From images of dissected tubules captured with the Leica M165 FC microscope, the tubules of interest were outlined, then total tubule area and pixel intensity histogram plots were obtained. Areas containing concretions were defined by setting the discrimination threshold to more than 3 standard deviations greater than the mean pixel intensity of control flies (*w1118*). Any area in tubules whose pixel intensity was above the threshold was considered to be a concretion. Accordingly, the percentage of the tubule lumen area occupied by concretions was calculated. For all image analysis, background intensity was subtracted.

### Collection of crystallized concretions

To obtain sufficient concretions for ICP-OES, μXRF, μXRD, and μXANES analysis, Malpighian tubules were dissected in sterile water and transferred to a pre-weighed centrifuge tube and sonicated (Ultrasonic Processor GEX130 sonicator, Cole-Parmer. Instruments, Vernon Hills, IL) at an amplitude of 20 for 5 seconds to separate tubule tissue from intraluminal concretions. After concretions settled by gravity for 10 minutes, the supernatant was removed and the concretions were washed 3 times in sterile water to remove remaining tubule tissue. Water was removed by aspiration and the concretions were allowed to dry in a negative pressure hood, which gave rise to approximately 1 mg of concretions from 150 *Da-GAL4*, *UAS-Xdh RNAi/+* flies fed on a high yeast diet for 2 days.

### Confocal microscopy

Concretions were manually dissected and collected into a microfuge tube containing 100% ethanol. Immediately prior to microscopy, stone suspensions were then vortexed on low speed for 10 seconds to detach debris and then centrifuged for 30 seconds at 500x*g*. The lowest 50μl was deposited onto a standard glass coverslip and the ethanol was allowed to evaporate. Concretions were then identified by size and shape using differential interference contrast (DIC) at 100X on a Zeiss LSM 710 Spectral Confocal Microscope. Broad-spectrum autofluorescence was then used for imaging using dual excitation at 488nm and 561nm and collecting emission over 580–700nm. Confocal optical slices of stones were collected at 2.4μm intervals in stack sizes ranging from 40–50 images depending on thickness of stone. Localization, measurements, and 3D reconstruction were determined using Bitplane Imaris Suite software.

### ICP-OES

Concretion elemental analysis was determined by inductively coupled plasma—optical emission spectroscopy (ICP-OES). Fly concretions were isolated by dissection, washed with distilled water, and dissolved in OmniTrace 70% HNO_3_ (VWR Scientific, Radnor, PA). Whole flies were collected, dried, and dissolved directly in OmniTrace 70% HNO_3_. All samples were then diluted with OmniTrace water to 5% HNO_3_ before being transferred via pneumatic nebulizer into a Vista Pro ICP (Varian Inc, Palo Alto, CA). Elemental values were calibrated by using National Institute of Standards and Technology-traceable standards. Cesium was used for ionization suppression and yttrium was used as an internal standard. Detection limits for queried elements ranged between 0.005–50 parts per million and coefficient of variation for interassay precision ranged between 1%-10%. Data were collected and analyzed using native software (ICP Expert).

### X-ray microprobe

μXRF mapping and μXANES measurements were performed on beamline 10.3.2 of the Advanced Light Source at Lawrence Berkeley National Lab (Berkeley, CA). For human Randall’s plaque studies, after appropriate institutional human research protocols were approved, renal papilla biopsies were taken from kidneys during surgery for removal of urinary stones. Fly concretions and Randall’s plaques were mounted onto X-ray transparent 100 nm thick Si3N4 windows (Silson Ltd). Maps were collected at 11k eV using a 3 x 3 μm beam spot size, 2 x 2 μm pixels, 150 ms dwell / pixel. Ca K-edge and Zn K-edge spectra were collected in QXAS mode on Randall’s plaque and *Da-GAL4*, *UAS-Xdh RNAi/+* concretions. Spectra were dead-time corrected, pre-edge background substracted and post-edge normalized using standard procedures [66]. Spectra were calibrated using an Sb foil (4132.2 eV). Least-square linear combination fitting the spectra was performed using a database of standards.

### Transmission Electron Microscopy

For transmission electron microscopy, dissected Malpighian tubules containing concretions were immersed in buffered aldehyde, stained with osmium tetroxide and then en bloc with uranyl acetate, and embedded in Embed 812 (Electron Microscopy Sciences, Hatfield, PA). 100 μm sections were collected on slot grids, post-stained with uranyl acetate and lead citrate, and viewed on a Philips Tecnai 12 electron microscope. Human Randall’s plaque samples were fixed in 2% paraformaldehyde and 2.5% glutaraldehyde in 0.150 M sodium cacodylate buffer for 14 hours, then rinsed in 0.1 M sodium cacodylate buffer and post-fixed in 2% osmium tetroxide and 0.8% potassium ferrocyanide in 0.1 M sodium cacodylate for 60 minutes. After rinsing and staining with 2% uranyl acetate for 30 minutes, dehydration was performed using a series of ethanol dilutions followed by submersion in 100% ethanol and infiltration with propylene oxide followed by Epon-812. Samples were embedded in epon-filled beem capsules and allowed to polymerize at 60°C for 72 h. Blocks were sectioned using a MT-7000 ultramicrotome (RMC Products, Boeckeler Instruments, Tucson, AR) at 60 nm and imaged on a Phillips Tecnai 12 transmission electron microscope.

### High performance liquid chromatography (HPLC)—mass spectrometry (MS)

HPLC was performed using a Shimadzu UFLC prominence system fitted with following modules: CBM-20A (Communication bus module), DGU-A_3_ (degasser), two LC-20AD (liquid chromatograph, binary pump), SIL-20AC HT (auto sampler) and connected to a Sepak GP-C18 column (4.6 x 250 mm, 5 μm, 120 Å) and a Sepak GP-C18 guard column (4.0 x 10 mm, 5 μm, 120 Å). MS was performed using a 4000 QTRAP LC-MS/MS mass spectrometer from AB SCIEX fitted with a TurboV ion source. AB SCiex’s Analyst v1.6.1 was used for all forms of data acquisition, development of HPLC method, and optimization of analyte-specific MRM (multiple reaction monitoring) transitions. PeakView v2.1 was used for the analysis of HPLC-MS and scheduled HPLC-MRM runs. For whole fly analysis, 5 flies for each genotype were flash-frozen over liquid nitrogen and subsequently homogenized ultrasonically using a Fisher Scientific’s 550 Sonic Dismembrator with 50 μL of a 6:4 mixture of methanol/water (*v/v*). Three 20 s pulses at amplitude setting 3 of the instrument (on ice) were sufficient to completely homogenize fly bodies. The homogenates were then vortexed for 5 times over a period of ~30 min (each 1 min long). Subsequently, the samples were centrifuged at 10,000 rpm for 10 min, the supernatant was filtered, and 3 μL of each was injected for scheduled HPLC-MRM analysis (*vide infra*) without any additional processing. For the analysis of fly concretions, samples were collected from 25 flies upon *Xdh* inhibition and extracted with 50 μL of a 6:4 mixture of methanol/water (*v/v*) and prepared for scheduled HPLC-MRM analysis (*vide infra*) as described above for whole flies.

### MRM optimization, HPLC-MS, and scheduled HPLC-MRM analyses

Optimization of analyte-specific MRM transitions, such as determination of suitable precursor and product ions and optimal MS parameters for each transition (Q_1_, precursor → Q_3_, product) were achieved by isocratic flow injection of 10 μM solution for each analyte. These standard solutions were prepared in 1:1 mixture of methanol/water (*v/v*) using commercially available synthetic compounds. For each analyte the transition that showed the highest signal-to-noise was used for quantification.

For HPLC separation, a solvent gradient of 0.1% acetic acid in water (aqueous)—acetonitrile (organic) was used with 1 mL/min flow rate, starting with an acetonitrile content of 1% for 3 min, which was increased to 40% over 12 min and then to 100% over 2 min and held at 100% for 4 min. The HPLC column was subsequently reconstituted to its initial condition (acetonitrile content of 1%) over the next 1 min and re-equilibrated for 5 min.

Metabolome extracts from whole flies and XDH concretions were analyzed by scheduled HPLC-MRM in both negative and positive ion modes. To develop the scheduled HPLC-MRM method, the MS was first operated in scanning mode for a mass range of *m/z* 100–300. 3 μL of a standard mixture containing 10 μM of individual purine metabolites was injected in each of two separate runs with the MS operating either in negative or positive mode. Source conditions for negative ionization mode were as follows: curtain gas (CUR) 20, nebulizer gas (GS1) 60, auxiliary gas (GS2) 50, ionspray voltage (IS) -4000 V, and source temperature (TEM) 450°C. Source conditions for positive ionization mode were as follows: CUR 20, GS1 60, GS2 50, IS 4500 V, and TEM 450°C. This step was undertaken to ascertain the HPLC retention times (RT) for the analytes of interest. Several such sets were acquired to compute analyte-specific variability in RT. Next, the MS was switched to operate in scheduled MRM mode, whereby the mass spectrometer acquired data for specific MRM transitions ±45 s around the computed RT for each analyte (S1 Table). Quantification was based on integration of analyte-specific peaks obtained from scheduled HPLC-MRM runs. Absolute amounts of individual analytes (in ng/fly) were calculated using response factors determined for commercial standards and making necessary adjustments for fly numbers and sample dilutions.

### Total RNA and cDNA preparation

Five to ten flies were anesthetized with CO_2_, which were immediately homogenized using a Kontes Microtube Pellet Pestle Rods with Motor in the tube containing 350 μl RLT media supplemented with 3.5 μl of β-mercaptoethanol (RNeasy Mini Kit, Qiagen, Valencia, CA, USA) and total RNAs were isolated according to the manufacturer’s protocol. After measuring total RNA concentration and confirming their quality by examining their absorbance at 260 and 280 nm with NanoDrop (Thermo Scientific, Wilmington, DE, USA), cDNA were converted from the total RNAs using QuantiTec Reverse Transcription Kit (Qiagen, Valencia, CA, USA).

### Quantitative polymerase chain reaction (qPCR)

Using cDNA as a template, qPCR with SensiFast SYBR NO-Rox Kit (Taunton, MA, USA) was performed using a Light Cycler 480 Real-Time PCR System (Roche Applied Science, Indianapolis, IN, USA) under the following conditions: 95°C, 5 seconds for denaturation, 55°C, 20 seconds for annealing and extension. For gene specific primer sets, β-tubulin (CG9277) F, ACA-TCC-CGC-CCC-GTG-GTC, R, AGA-AAG-CCT-TGC-GCC-TGA-ACA-TAG. CG3994, F, tgg-ttt-tta-tga-ttg-ccg-aaa, R, aat-gag-cgg-cgt-ctg-ttg, CG17723, F, cat-cga-ggc-ttg-caa-aag-a, R, gac-aag-cag-ttc-agg-ctc-gt, CG11163, F, aat-taa-ttc-aaa-cga-gca-taa-gat-tg, R, tat-cct-cgt-gtg-cca-gct-c. CG7642, F, tgg-tga-ctt-ccc-act-gga-g, R, ggt-tcg-ggt-att-tca-agc-ag were used. The specificity of amplicons was verified with a melting curve analysis and the messenger levels were normalized using β-tubulin as an internal control and calculated according to the ΔΔCt method.

## Supporting Information

S1 Fig Zn transporter RNAi lines significantly reduced the corresponding endogenous Zn transporter mRNA levels. (A to D) cDNAs converted from total RNA isolated from *Da-GAL4/+*, *Da-GAL4*, *Xdh RNAi /+*, *Da-GAL4*, *Xdh RNAi /CG3994 RNAi*, *Da-GAL4*, *Xdh RNAi /CG17723 RNAi and Da-GAL4*, *Xdh RNAi /CG11163 RNAi* male flies fed 5% YE for 2 days were subjected to qPCR. For each genotype, the relative endogenous level of each Zn transporter (*CG3994*, *CG17723*, *CG11163*) and xanthine dehydrogenase were calculated with ß-tubulin as an internal control. Simultaneous knockdown of zinc transporter expression did not result in reversal of *Xdh* inhibition. *Da* = *Da-GAL4*, *Xdh* = *UAS-Xdh RNAi* (**p<0.01, ***p<0.001, one way ANOVA with Bonferroni post-hoc test for (D) compared to Da/+, Student’s t-test for all others, n = 3). Data shown are the mean ± SEM.
(TIF)

S2 Fig Concretion accumulation occurs in a similar fashion in two different RNAi lines inhibiting *Xdh* and is not affected by aging. (A to C) Representative images of tubules dissected from flies after being fed a high yeast diet for 3 days demonstrate that, compared to control flies seen in (A), inhibition of two different RNAi lines results in similar rates of concretion formation within the lumen of the tubule. Both lines were obtained from the Vienna *Drosophila* RNAi Center where *Xdh(a)* is transformant ID number 25172 inserted on chromosome 3 (used for all the described experiments), and the confirmatory *Xdh(b)* is transformant ID number 106995 inserted on chromosome 2. (D to F) In control flies fed on high (5% YE) and low (0.5% YE) yeast diets, minimal increase in the accumulation of concretions within the tubule of the Malpighian tubule is seen when comparing representative dissection specimens from day of life 15, 35, or 45 respectively.
(TIF)

S3 Fig Allopurinol feeding enhances stone formation in fly Malpighian tubules. (A) *Da-GAL4 /+* control flies exhibit minimal tubule concretions in the absence of allopurinol. (B) Feeding control flies on 5% YE food supplemented with 500 μM allopurinol for 14 days, tubule concretions can be seen accumulated to a similar level compared to *Xdh*-silenced flies. Allopurinol is a pharmacologic xanthine dehydrogenase inhibitor.
(TIF)

S4 Fig Fourier Transform Infrared Spectroscopy (FTIR) analysis of *Drosophila* concretion samples. FTIR, the traditional method by which kidney stones are analyzed clinically, was performed on Malpighian tubule intraluminal concretions collected from *Da-Gal4*, *UAS-Xdh RNAi/+* flies. This analysis demonstrated that these concretions exhibit a similar spectroscopic appearance compared to pure xanthine control material.
(TIF)

S5 Fig Concretion samples removed from the tubular lumen demonstrate minimal cellular contamination. (A and B) Malpighian tubule intraluminal concretions were collected from *Da-Gal4*, *UAS-Xdh RNAi/+* flies. Dissected samples were sonicated and then washed to isolate stones from tubule cells and debris. Brightfield (BF) microscopy and DAPI staining demonstrate the presence of cellular contamination after sonication in only the unwashed samples seen in (A), as indicated by the yellow arrows.
(TIF)

S6 Fig Two additional zinc transporter lines demonstrate reduction in concretion formation and rescue of survivorship upon inhibition of xanthine dehydrogenase. (A) On 5% YE, simultaneous inhibition of xanthine dehydrogenase and three different zinc transporters (*Xdh* x *CG11163*, *Xdh* x *CG17723*, and *Xdh* x *CG3994*) resulted in significantly decreased concretion accumulation compared to *Da-GAL4*, *UAS-Xdh RNAi /+* flies (*Xdh* x *W1118*) (***p <0.001, one way ANOVA with Bonferroni post-hoc test, n = 14–43). (B) Simultaneous inhibition of the same three zinc transporters with xanthine dehydrogenase rescued survivorship when compared with *Da-GAL4*, *UAS-Xdh RNAi /+* animals on 5% YE (logrank test, n = 76–97).
(TIF)

S7 Fig Zn transporter inhibition does not alter whole fly Ca and Mg levels. (A to B) Upon simultaneous RNAi inhibition of xanthine dehydrogenase and Zn transporters, male flies were fed 5% YE. After 2 days, flies were anesthetized with CO_2_, their body weights measured and whole flies homogenized and analyzed with ICP-OES to examine whole-fly levels of Zn (Fig 3B), Ca and Mg and compared to *Da-GAL4*, *UAS-Xdh RNAi /+* controls. Data shown are the mean ± SEM.
(TIF)

S8 Fig Titration of TPEN concentration demonstrates dose dependent effects on inhibition of stone formation. (A) Survival curves of *Da-GAL4*, *UAS-Xdh RNAi /+* (left panel) and *Da-GAL4/+* (right panel) male flies fed 5% YE supplemented with different concentration of TPEN (**p<0.01, logrank test [5% YE vs 5% YE + 1mM TPEN], n = 21–132). (B) *Da-GAL4*, *UAS-Xdh RNAi /+* male flies were fed 5% YE with and without TPEN at two different doses. After two days of TPEN supplementation, tubules were dissected and concretion formation quantified (**p<0.01, one way ANOVA with Bonferroni post-hoc test, n = 21–48). Data shown are the mean ± SEM.
(TIF)

S9 Fig Inhibition of three zinc transporters from the ZnT family demonstrates only modest effect on lifespan. (A to C) Using the drug-inducible ubiquitous driver *Act5c-GAL4*, inhibition of each of the ZnT transporters demonstrates no significant extension of lifespan that would account for the rescue of survivorship seen in Fig 3 or S6 Fig In the cases of *UAS-CG3994* and *UAS-CG17723*, neither the median nor the maximum lifespan were changed in either low or high yeast feeding conditions. In the case of *UAS-CG11163* pictured in (C), the maximum but not the median lifespans were reduced in both feeding conditions. 0.5% YE = low yeast. 5% YE = high yeast. (-) indicates the absence of RU-486 and (+) indicates its presence in the food.
(TIF)

S1 Table MRM conditions for purines. (TIF)
