# Supplementary figures and images for: A Drosophila Model Identifies a Critical Role for Zinc in Mineralization for Kidney Stone Disease
Source: PLoS One. 2015 May 13;10(5):e0124150. doi: 10.1371/journal.pone.0124150 (PMC4430225; doi:10.1371/journal.pone.0124150)

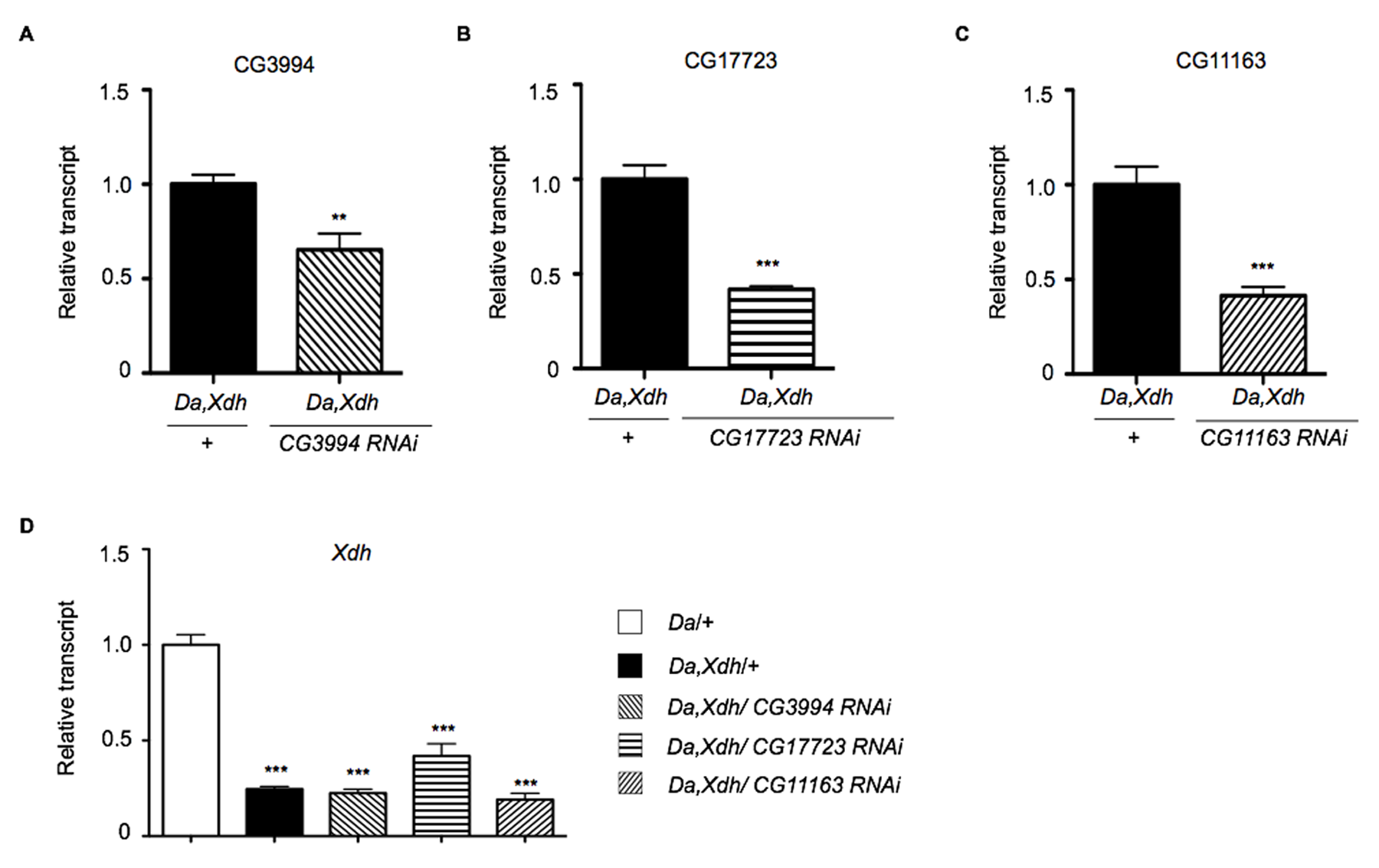

Supplement: S1 Fig — (A to D) cDNAs converted from total RNA isolated from Da-GAL4/+, Da-GAL4, Xdh RNAi /+, Da-GAL4, Xdh RNAi /CG3994 RNAi, Da-GAL4, Xdh RNAi /CG17723 RNAi and Da-GAL4, Xdh RNAi /CG11163 RNAi male flies fed 5% YE for 2 days were subjected to qPCR. For each genotype, the relative endogenous level of each Zn transporter (CG3994, CG17723, CG11163) and xanthine dehydrogenase were calculated with ß-tubulin as an internal control. Simultaneous knockdown of zinc transporter expression did not result in reversal of Xdh inhibition. Da = Da-GAL4, Xdh = UAS-Xdh RNAi (**p<0.01, ***p<0.001, one way ANOVA with Bonferroni post-hoc test for (D) compared to Da/+, Student’s t-test for all others, n = 3). Data shown are the mean ± SEM. (TIF) [file pone.0124150.s001.tif]

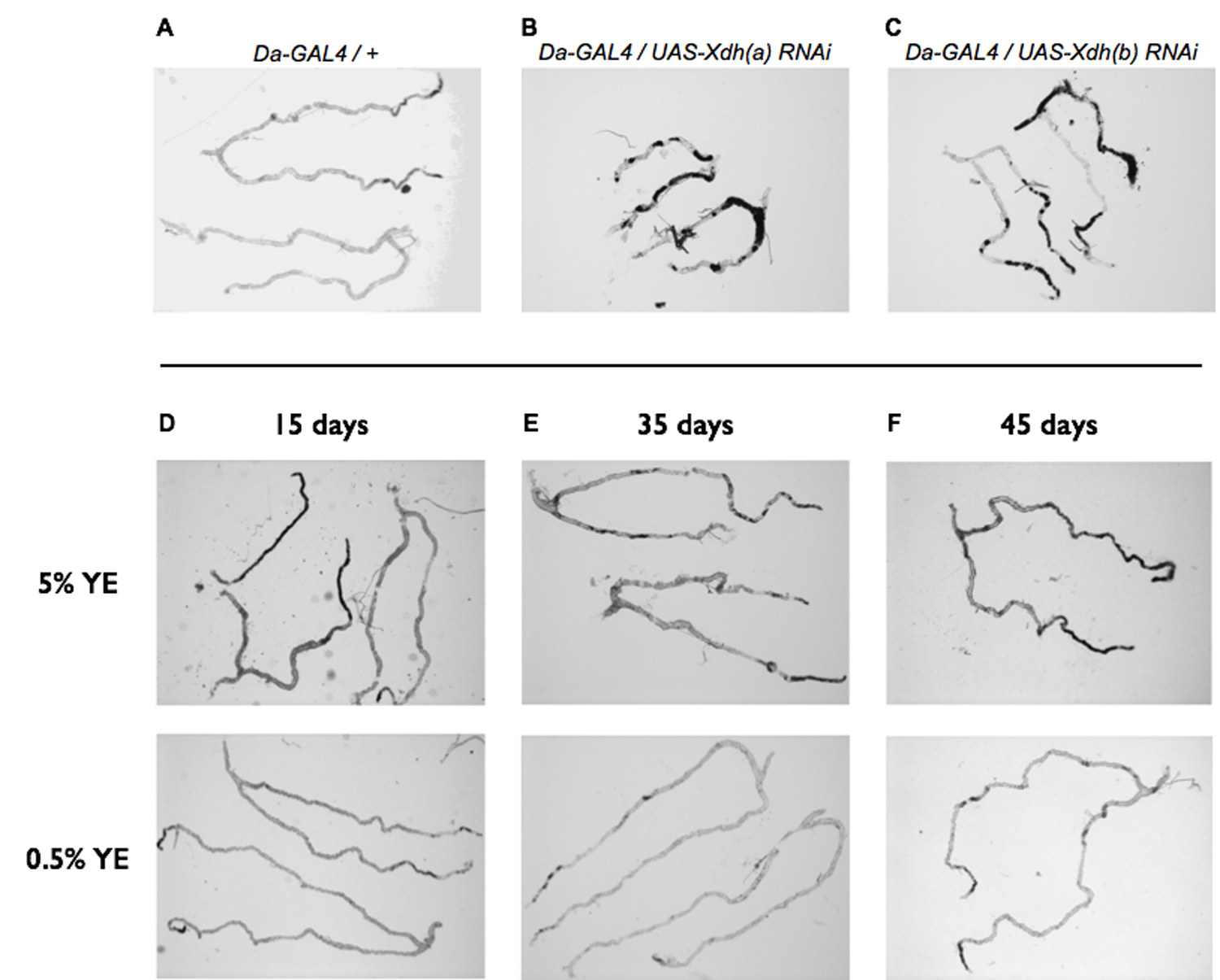

Supplement: S2 Fig — (A to C) Representative images of tubules dissected from flies after being fed a high yeast diet for 3 days demonstrate that, compared to control flies seen in (A), inhibition of two different RNAi lines results in similar rates of concretion formation within the lumen of the tubule. Both lines were obtained from the Vienna Drosophila RNAi Center where Xdh(a) is transformant ID number 25172 inserted on chromosome 3 (used for all the described experiments), and the confirmatory Xdh(b) is transformant ID number 106995 inserted on chromosome 2. (D to F) In control flies fed on high (5% YE) and low (0.5% YE) yeast diets, minimal increase in the accumulation of concretions within the tubule of the Malpighian tubule is seen when comparing representative dissection specimens from day of life 15, 35, or 45 respectively. (TIF) [file pone.0124150.s002.tif]

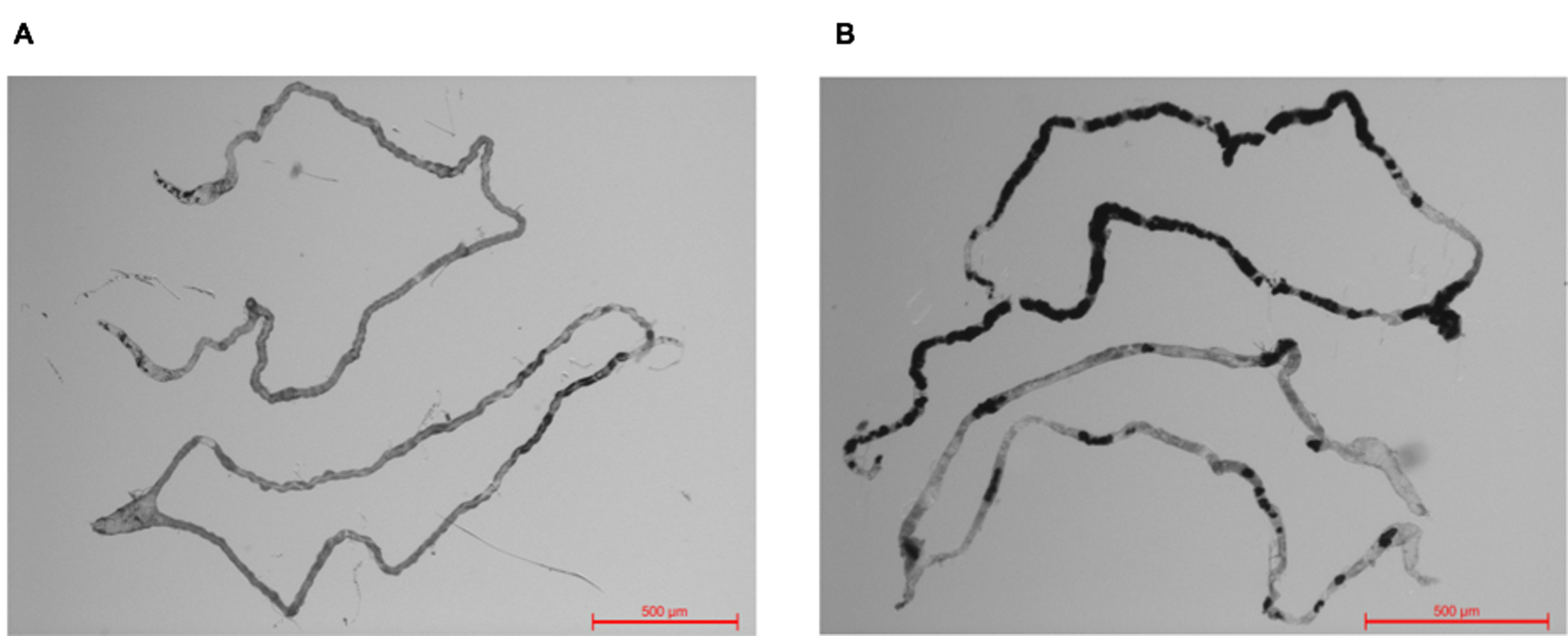

Supplement: S3 Fig — (A) Da-GAL4 /+ control flies exhibit minimal tubule concretions in the absence of allopurinol. (B) Feeding control flies on 5% YE food supplemented with 500 μM allopurinol for 14 days, tubule concretions can be seen accumulated to a similar level compared to Xdh-silenced flies. Allopurinol is a pharmacologic xanthine dehydrogenase inhibitor. (TIF) [file pone.0124150.s003.tif]

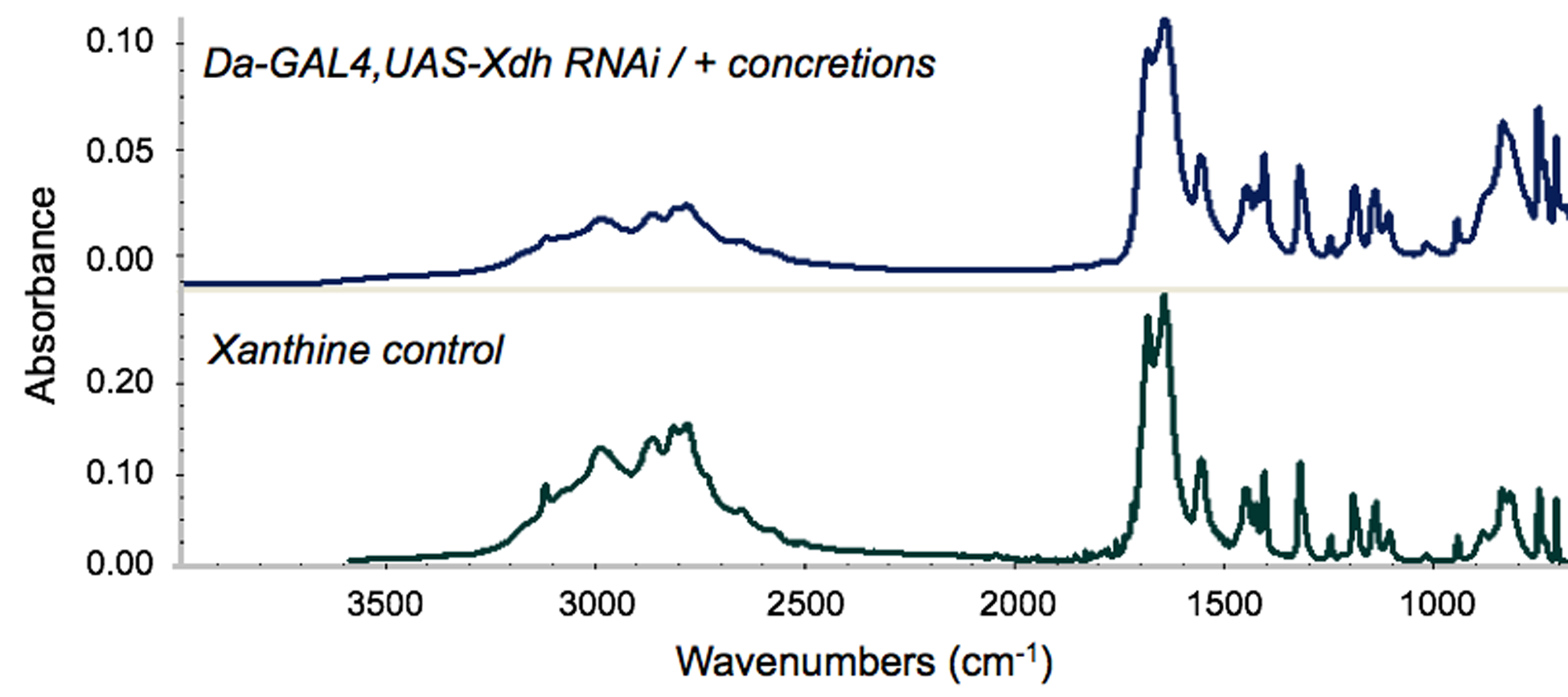

Supplement: S4 Fig — FTIR, the traditional method by which kidney stones are analyzed clinically, was performed on Malpighian tubule intraluminal concretions collected from Da-Gal4, UAS-Xdh RNAi/+ flies. This analysis demonstrated that these concretions exhibit a similar spectroscopic appearance compared to pure xanthine control material. (TIF) [file pone.0124150.s004.tif]

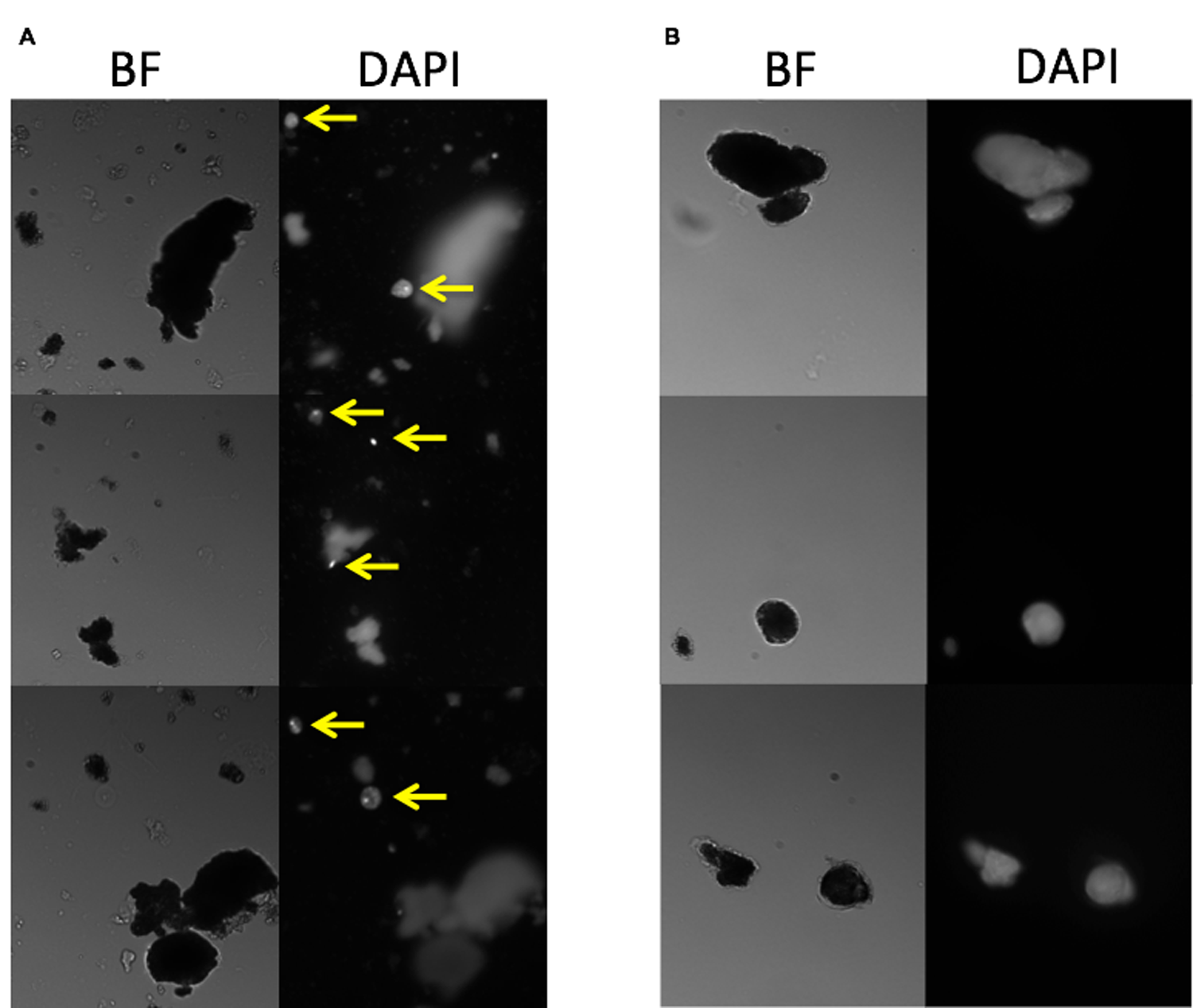

Supplement: S5 Fig — (A and B) Malpighian tubule intraluminal concretions were collected from Da-Gal4, UAS-Xdh RNAi/+ flies. Dissected samples were sonicated and then washed to isolate stones from tubule cells and debris. Brightfield (BF) microscopy and DAPI staining demonstrate the presence of cellular contamination after sonication in only the unwashed samples seen in (A), as indicated by the yellow arrows. (TIF) [file pone.0124150.s005.tif]

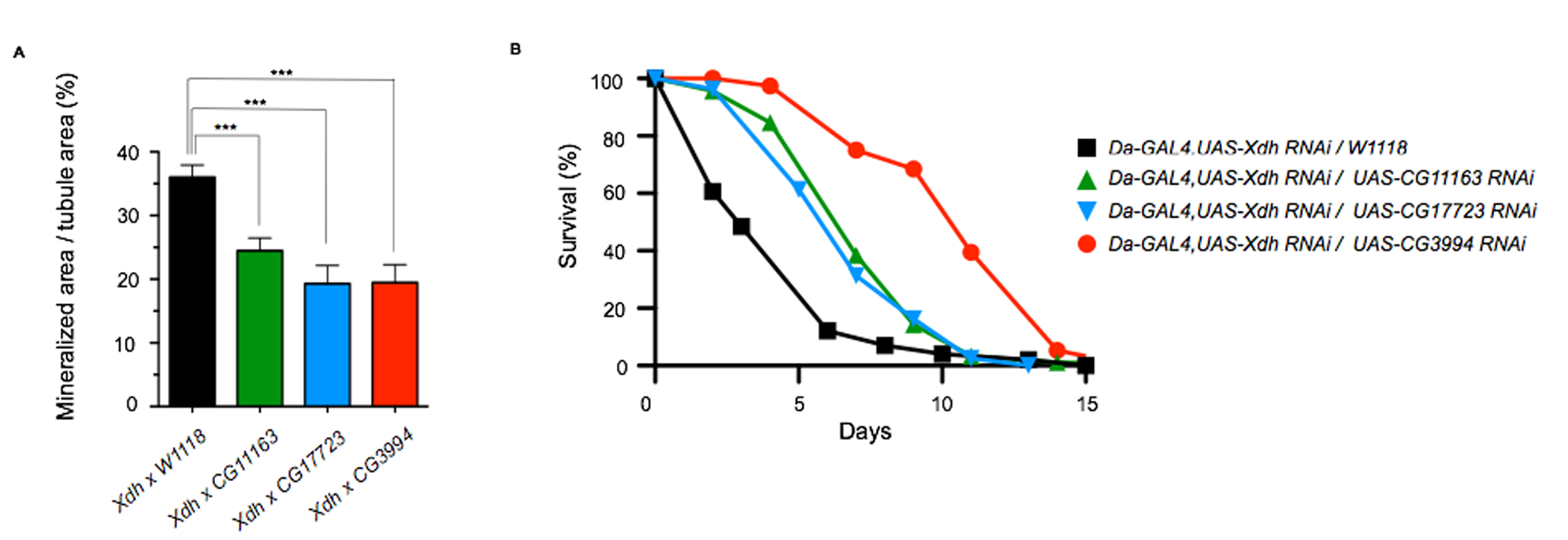

Supplement: S6 Fig — (A) On 5% YE, simultaneous inhibition of xanthine dehydrogenase and three different zinc transporters (Xdh x CG11163, Xdh x CG17723, and Xdh x CG3994) resulted in significantly decreased concretion accumulation compared to Da-GAL4, UAS-Xdh RNAi /+ flies (Xdh x W1118) (***p <0.001, one way ANOVA with Bonferroni post-hoc test, n = 14–43). (B) Simultaneous inhibition of the same three zinc transporters with xanthine dehydrogenase rescued survivorship when compared with Da-GAL4, UAS-Xdh RNAi /+ animals on 5% YE (logrank test, n = 76–97). (TIF) [file pone.0124150.s006.tif]

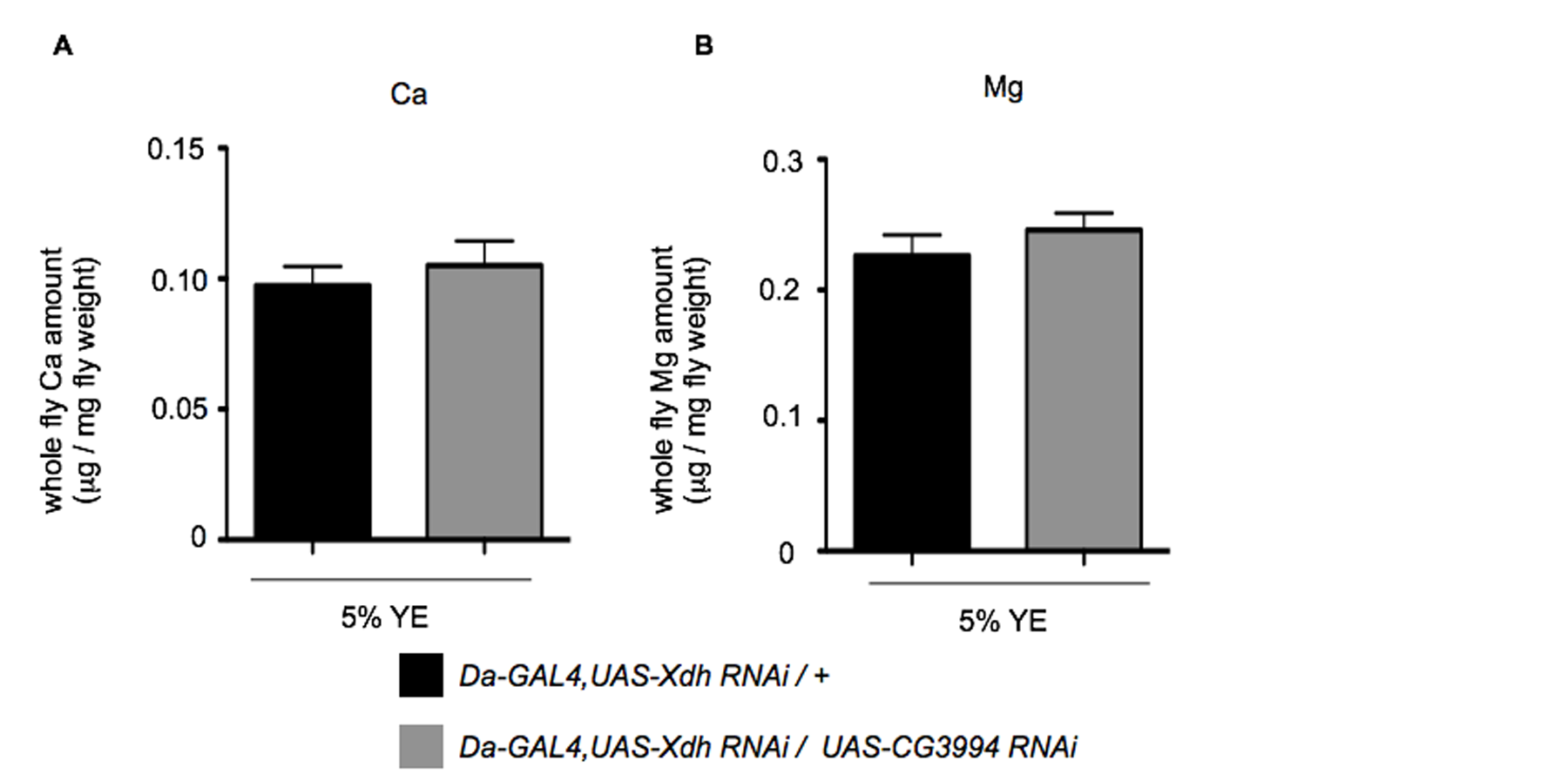

Supplement: S7 Fig — (A to B) Upon simultaneous RNAi inhibition of xanthine dehydrogenase and Zn transporters, male flies were fed 5% YE. After 2 days, flies were anesthetized with CO2, their body weights measured and whole flies homogenized and analyzed with ICP-OES to examine whole-fly levels of Zn (Fig 3B), Ca and Mg and compared to Da-GAL4, UAS-Xdh RNAi /+ controls. Data shown are the mean ± SEM. (TIF) [file pone.0124150.s007.tif]

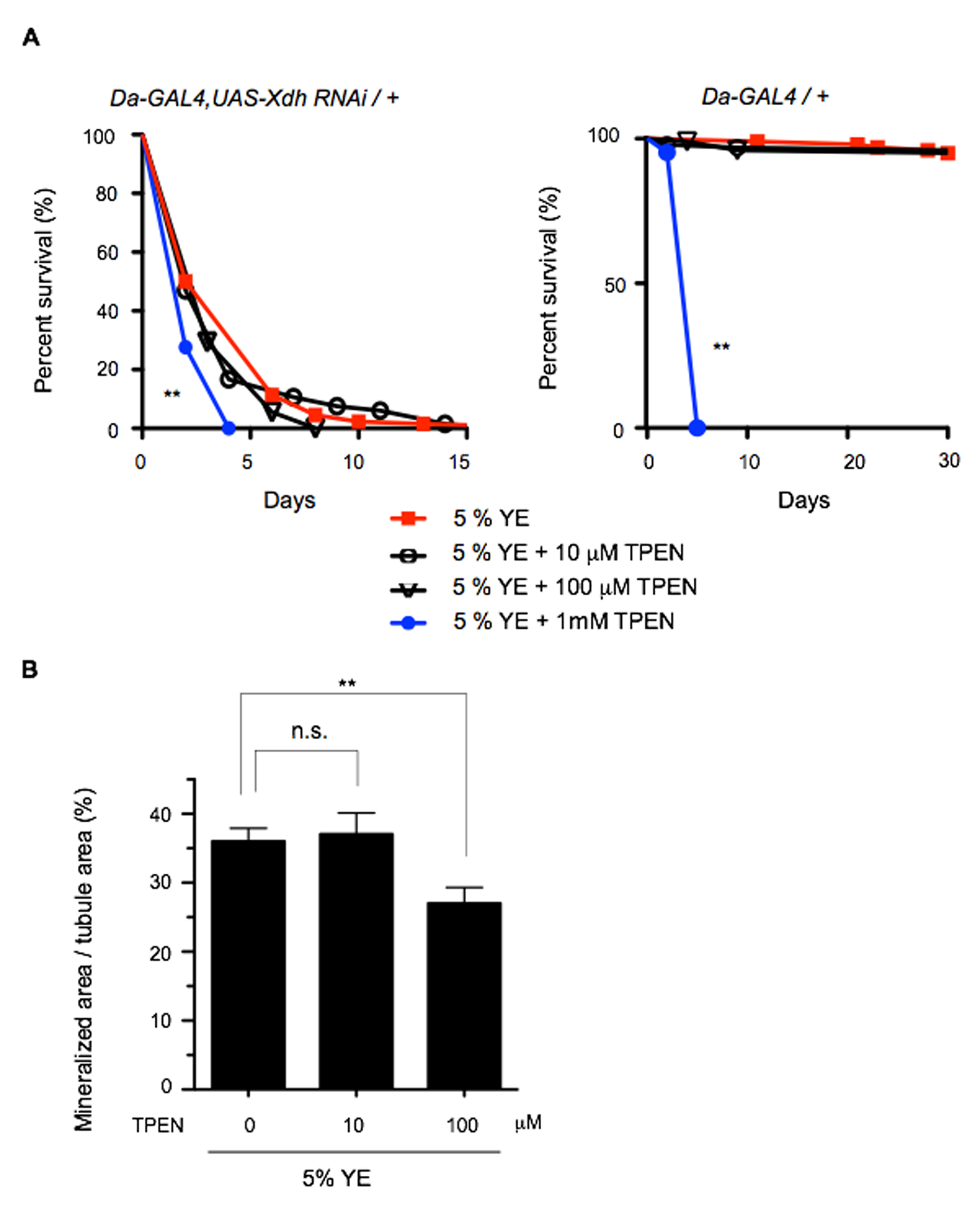

Supplement: S8 Fig — (A) Survival curves of Da-GAL4, UAS-Xdh RNAi /+ (left panel) and Da-GAL4/+ (right panel) male flies fed 5% YE supplemented with different concentration of TPEN (**p<0.01, logrank test [5% YE vs 5% YE + 1mM TPEN], n = 21–132). (B) Da-GAL4, UAS-Xdh RNAi /+ male flies were fed 5% YE with and without TPEN at two different doses. After two days of TPEN supplementation, tubules were dissected and concretion formation quantified (**p<0.01, one way ANOVA with Bonferroni post-hoc test, n = 21–48). Data shown are the mean ± SEM. (TIF) [file pone.0124150.s008.tif]

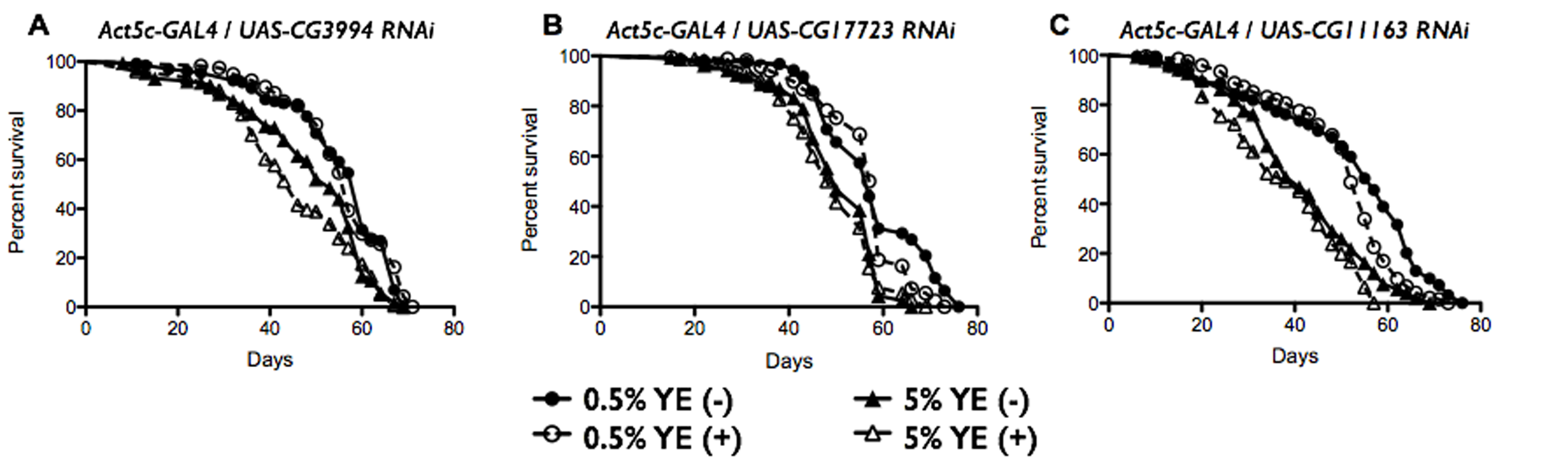

Supplement: S9 Fig — (A to C) Using the drug-inducible ubiquitous driver Act5c-GAL4, inhibition of each of the ZnT transporters demonstrates no significant extension of lifespan that would account for the rescue of survivorship seen in Fig 3 or S6 Fig In the cases of UAS-CG3994 and UAS-CG17723, neither the median nor the maximum lifespan were changed in either low or high yeast feeding conditions. In the case of UAS-CG11163 pictured in (C), the maximum but not the median lifespans were reduced in both feeding conditions. 0.5% YE = low yeast. 5% YE = high yeast. (-) indicates the absence of RU-486 and (+) indicates its presence in the food. (TIF) [file pone.0124150.s009.tif]

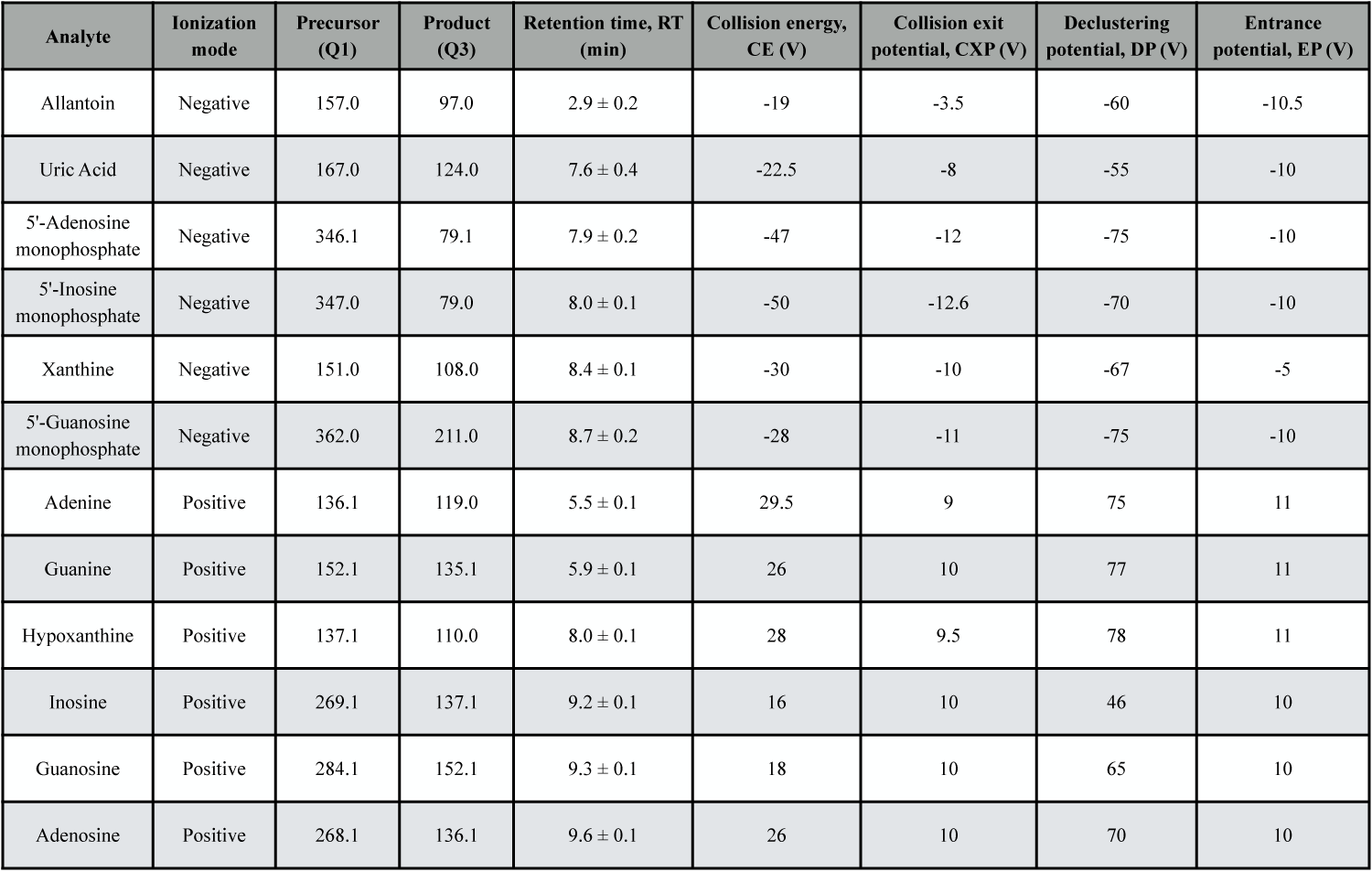

Supplement: S1 Table — (TIF) [file pone.0124150.s010.tif]
